# Lymphatic microvessel density as a predictive marker for the recurrence time of pterygium: A three-year follow-up study

**Published:** 2013-01-28

**Authors:** Haotian Lin, Lixia Luo, Shiqi Ling, Wan Chen, Zhaochuan Liu, Xiaojian Zhong, Changrui Wu, Weirong Chen, Yizhi Liu

**Affiliations:** 1 State Key Laboratory of Ophthalmology, Zhongshan Ophthalmic Center, Sun Yat-sen University, Guangzhou, China; 2 Department of Ophthalmology, The Third Affiliated Hospital of Sun Yat-sen University, Guangzhou, China

## Abstract

**Purpose:**

To investigate whether lymphatic microvessel density (LMVD) could be used as a predictive marker for the recurrence time of pterygia.

**Methods:**

This was a prospective case series study. Ninety-six patients with unilateral eye primary nasal pterygia were included. The patients were clinically evaluated to grade the severity of their pterygia (32 were Grade 1, 29 were Grade 2, and 35 were Grade 3) before they underwent bare sclera resection with the use of mitomycin C. Excised tissues from the 96 patients and the ten normal nasal conjunctiva obtained from age-matched donor eyeballs (controls) were immunostained with LYVE-1 and CD31 monoclonal antibodies to evaluate LMVD and blood microvessel density (BMVD). The patients were followed up for three years or until pterygium recurrence was identified, which was defined as fibrovascular regrowth past the limbus in a previously compromised area. The recurrence time (RT) for a pterygium was calculated, and its relationship with LMVD and/or BMVD was statistically analyzed.

**Results:**

In total, there were 24 cases of pterygium recurrence. The recurrence rate of Grade 1 was 28.1% (9/32), Grade 2 was 24.1% (7/29), and Grade 3 was 22.9% (8/35), as classified in the primary pterygium (p>0.05); the overall recurrence rate was 25% (24/96) for all patients during the three-year follow-up. In the tissue analysis, there were a small number of CD31 (+), LYVE-1(-) BMVD and only a few CD31 (weak), LYVE-1(+) LMVD in the ten normal nasal conjunctiva tissues. BMVD and LMVD increased significantly in the pterygium tissue compared to the control tissue and were significantly correlated with the width and area of pterygium in Grades 1–3 (all p values <0.05). RT was not correlated with BMVD or pterygium grade, but LMVD was significantly and negatively correlated with RT within each group and in the total patient cohort. Furthermore, we determined that an LMVD greater than 20 in the surgical specimens predicted pterygium recurrence.

**Conclusions:**

The LMVD of surgical specimens is an independent risk factor and a valuable predictive factor for the recurrence time of pterygia.

## Introduction

A pterygium is a neoformation characterized by the encroachment of a fleshy fibrovascular tissue from the bulbar conjunctiva onto the cornea [1]. The pathogenesis of pterygia has intrigued researchers for centuries, but it is still not completely understood. Studies have shown an increasing prevalence of pterygia with increasing proximity to the equator, secondary to increased exposure to ultraviolet radiation [2-5]. Other investigators have proposed neoplastic factors, focusing on the p53 tumor suppressor gene [6,7], while some believe tear film abnormalities and allergic factors are significant contributors [8].

More recently, immunopathologic mechanisms have been studied to determine their roles in the pathogenesis of pterygia, including humoral immunity [9-11], cellular immunity, and hypersensitivity [12]. Blood vessels play an important role in the formation and progression of pterygia [13-15]. In our previous study, we demonstrated with enzyme histochemistry [16] and immunohistochemistry [17] that lymphatic microvessel density (LMVD) increased dramatically with the severity of pterygia. Furthermore, we showed that the transient upregulation of vascular endothelial growth factor-C may be responsible for lymphangiogenesis in this setting [17]. Therefore, we hypothesize that pathological angiogenesis and lymphangiogenesis comprise two arms of a potential “immune reflex” that could lead to an abnormal immune response [18-20], thus resulting in the pathogenesis of a primary pterygium or the accelerated recurrence of a pterygium, similar to the role angiogenesis and lymphangiogenesis play in the inflammatory cornea [21,22], the rejected transplanted cornea [23,24], and the acceleration of corneal rejection [25].

The goals of the present study were to determine whether LMVD and/or blood microvessel density (BMVD) are related to the recurrence time of pterygia, and whether they could be used as predictive markers for the recurrence of pterygia. Findings from this study may provide additional evidence contributing to our understanding of immunological process associated with lymphangiogenesis that can be instrumental in the pathogenesis of pterygia.

## Methods

### Inclusion criteria

This was a prospective case study. Patients under 65 years old who planned to receive surgery for their primary unilateral eye nasal pterygia were included in the study. The primary unilateral eye nasal pterygium had invaded the cornea with an apex past the limbus of at least 1 mm. The visual acuity of the patient was influenced, or he or she felt uncomfortable with the pterygium and required surgery. No systemic immune diseases were found, and no immunosuppressant was used preoperatively. No history of ocular surgery, ocular injury, or other ocular diseases was identified. At the same time, ten nasal epibulbar conjunctiva segments near the limbus, excised from ten age-matched control donor eyeballs that had been identified without ocular diseases or systemic immune diseases, were used as the control samples. The protocol and informed consent forms were reviewed and approved by the Institutional Review Board/Ethics Committee of the Sun Yat-sen University, and a written informed consent form was given to each study participant. The study was conducted in accordance with the Declaration of Helsinki and the ethical standards of the local ethics committee. All of the participants signed consent forms and were followed according to our schedule.

### Preoperative evaluation

**Figure 1:**
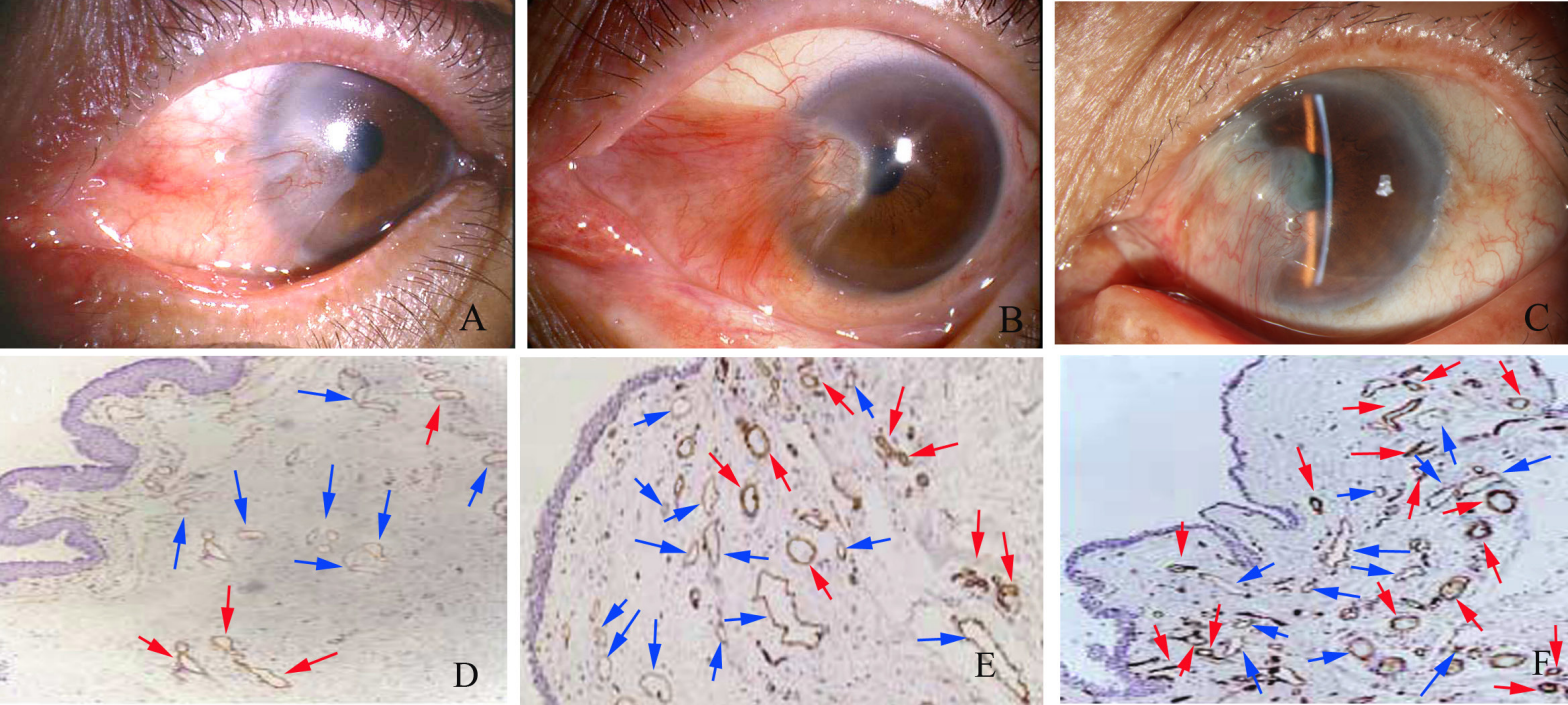
Slit-lamp photos of different grades of pterygium and results of lymphatic vessel endothelial hyaluronan receptor 1 immunohistochemistry for the three grades of pterygia: **A** and **D**: Grade 1; **B** and **E**: Grade 2; **C** and **E**: Grade 3. Microvessels were dramatically increased in Grades 2 and 3 pterygia. Lymphatic vessel endothelial hyaluronan receptor 1 (LYVE-1) was positive for lymphatic vessels but negative for blood vessels. Red arrows point to lymphatic vessels and blue arrows, blood vessels (original magnification=100X).

Clinical evaluations were performed according to the grading systems described by Awdeh [26]. Briefly, pterygia was preoperatively graded on a scale of 1–3: 1+ (mild), 2+ (moderate), and 3+ (severe), based on objective signs, including conjunctival congestion and edema, relative thickness of the fibrovascular lesion, and general eye redness (Figure 1A–C). The size of the pterygium, including the horizontal extension onto the cornea from the limbus and the width of the base at the limbus, was measured (in millimeters) using a slit lamp, and the total area was calculated (Table 1).

**Table 1:** Characteristics of patients with pterygium in the study.

Grades of pterygium	Grade 1	Grade 2	Grade 3
Number of patients (eyes)	32	29	35
Age (years)	54.2±6.43	57.8±5.21*	56.3±5.92*+
Female/Male	18/14	16/13	20/15
Extension of pterygium (mm)	1.71±0.22	2.12±0.14 #	3.05±0.53 # &
Width of pterygium (mm)	3.82±0.34	3.99±0.57*	4.13±0.55 # &
Area of pterygium (mm2)	6.90±1.13	7.28±1.54*	9.92±1.95 # &

Compared with grade 1, * p>0.05; # p<0.05; Compared with grade 2, &p<0.05, +p>0.05

### Surgical and postoperative protocol

For all included patients, bare sclera resection surgeries with mitomycin C were performed according to standard methods [27,28] by the same surgeon (Dr. Lin), and the patients received the same agents after each surgery. The detailed postoperative regimen included the following: Ofloxacin eye ointment (Santen Pharmaceutical Co., Ltd., Osaka, Japan) applied to the eye before corneal epithelial healing, compound tobramycin eye drops (Alcon, Texas, TX; 0.3% tobramycin + 0.1% dexamethasone) applied four times a day for 1 month after corneal epithelial healing, and compound tobramycin eye ointment (Alcon; 0.3% tobramycin + 0.1% dexamethasone) applied every night for 1 month after corneal epithelial healing.

### Immunohistochemistry

After being fixed in 10% neutral formalin for 24 h, embedded in paraffin, serially sectioned (4 μm thickness), and rehydrated with graded ethanol-water mixtures, the excised conjunctiva segments were washed with distilled water. Endogenous peroxidase activity was blocked after the segments were incubated with 30 ml/L hydrogen peroxidase for 20 min. For antigen retrieval, tissue sections were autoclaved at 121 °C in 10 mmol/l citrate buffer (pH 6.0) for 10 min. Next, the sections were allowed to cool at room temperature for 30 min. Subsequently, sections were incubated for 3 h with either a mouse antihuman LYVE-1 monoclonal antibody (R&D Systems, Rochester, MN) or a mouse antihuman CD31 monoclonal antibody (R&D Systems), and a biotin-labeled rabbit antimouse immunoglobulin antibody was used as the secondary antibody. The streptavidin biotin-peroxidase complex was used as the immunodetection system. The slides were visualized for peroxidase activity using diaminobenzidine (DAB) and counterstained with hematoxylin.

### Lymphatic microvessel density and blood microvessel density

LMVD and BMVD of human excised tissues were evaluated independently by two observers without prior knowledge of the experimental details, and the tests were repeated once. The CD31 (+), LYVE-1(-) vessels of sections were identified as blood vessels; whereas the CD31 (weak), LYVE-1(+) vessels were classified as lymphatic vessels (Figure 1D–F). Each sample was cut into 40 sections. Next, the sections were analyzed using standard light microscopy (Nikon, Eclipse 200, Tokyo, Japan). Under 100X magnification (0.78 mm^2^), the five most lymphovascularized areas were identified, and the immunostained lymphatic vessels were counted. Only vessels exhibiting typical morphology (containing lumen) were considered lymphatic microvessels. The LMVD for each case was expressed as the mean value (total number of vessels in 200 microscopic fields/200). Similarly, to calculate BMVD, all blood vessels in 200 fields from the 40 sections were summed and divided by 200.

### Follow-up protocol

**Figure 2:**
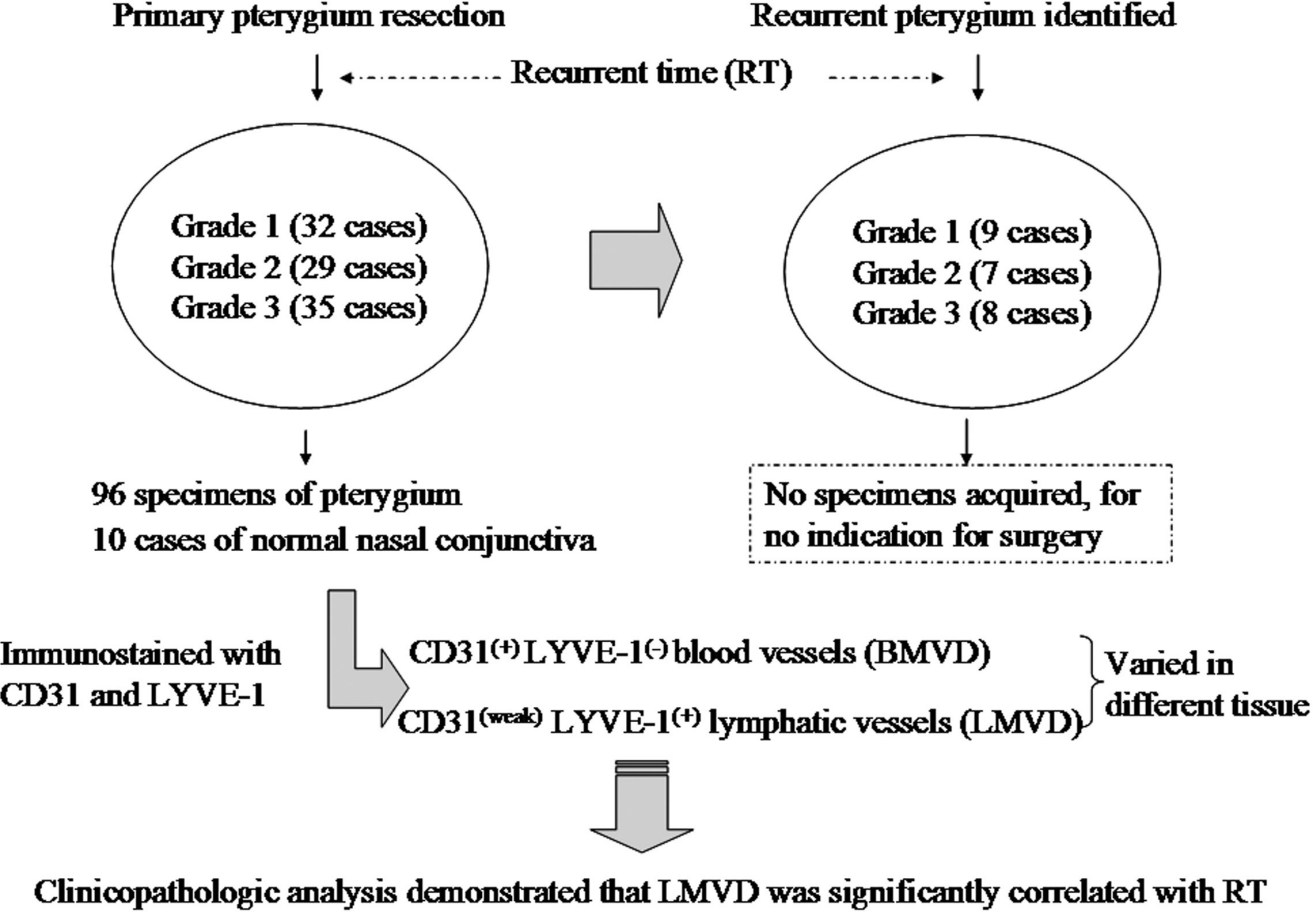
Study protocol, definition of recurrence time, and follow-up results.

After surgery, follow-up with each patient was performed every four weeks for three years or until pterygium recurrence was identified, as defined by fibrovascular regrowth past the limbus in a previously compromised area. The pterygium recurrence time (RT) was calculated as the duration between the time of the primary pterygium resection and the time of pterygium recurrence (Figure 2).

### Statistics

Significant differences were determined using the paired Student *t* test for normally distributed data and analysis of variance (ANOVA) for non-normally distributed data using SPSS software (version 17.0; SPSS, Inc., Chicago, IL). A multivariate model for RT was developed. The values are presented as the mean±standard deviation (SD). Differences were considered significant when p<0.05.

## Results

### Patients’ characteristics and grades of pterygium

Ninety-six patients (42 men and 54 women) from January 2006 to June 2010 who met the inclusion criteria and finished the follow-up were included in the study. Of the 96 subjects, preoperative Grade 1 pterygium was found in 32 (33.3%) patients, preoperative Grade 2 in 29 (30.2%) patients, and preoperative Grade 3 in 35 (36.5%) patients. The extension of the pterygium onto the cornea ranged from 1.2 mm to 4.7 mm with a mean of 2.32±0.81 mm. The width ranged from 1.6 mm to 7.1 mm with a mean of 3.98±0.95 mm. The total area ranged between 1.3 mm^2^ and 14.4 mm^2^ with a mean of 8.12±2.63 mm^2^ (Table 1).

### Blood and lymphatic vessels in pterygium

Immunohistochemical staining for LYVE-1 and CD31 was performed on serial sections of human pterygium tissue. Because CD31 stains blood and lymphatic vessels, and LYVE-1 stains only the lymphatic endothelium [29,30], we identified and distinguished blood and lymphatic vessels simultaneously in histological sections. Compared to blood vessels, lymphatic vessels had a relatively larger lumen and did not contain erythrocytes. Our data showed that there was a small number of CD31(+), LYVE-1(-) blood vessels, but only a few CD31(weak), LYVE-1(+) lymphatic vessels in normal epibulbar conjunctiva segments [16,17]. Lymphatic vessels were mildly increased in preoperative Grade 1 pterygia, but were dramatically increased in preoperative Grades 2 and 3 pterygia (Figure 1D–F and Table 2). However, relative to lymphatic vessels, there were greater blood vessel densities in preoperative Grade 2 pterygia, but the BMVD decreased in preoperative Grade 3.

**Table 2:** LMVD and BMVD identified in pterygium and control conjunctiva tissue.

Grades of pterygium	Number of cases	LMVD	BMVD
Grade 1	32	12.75±8.97*	19.66±5.15
Grade 2	29	15.1±7.43 *	36.52±9.12*
Grade 3	35	27.29±8.45 *	29.31±9.27 *
Normal conjunctiva	10	7.7±4.47	18.6±4.22

LMVD=Lymphatic Micro-Vessel Density; BMVD=Blood Micro-Vessel Density; Compared with normal conjunctiva, * p<0.05.

### Pterygium recurrence time

**Figure 3:**
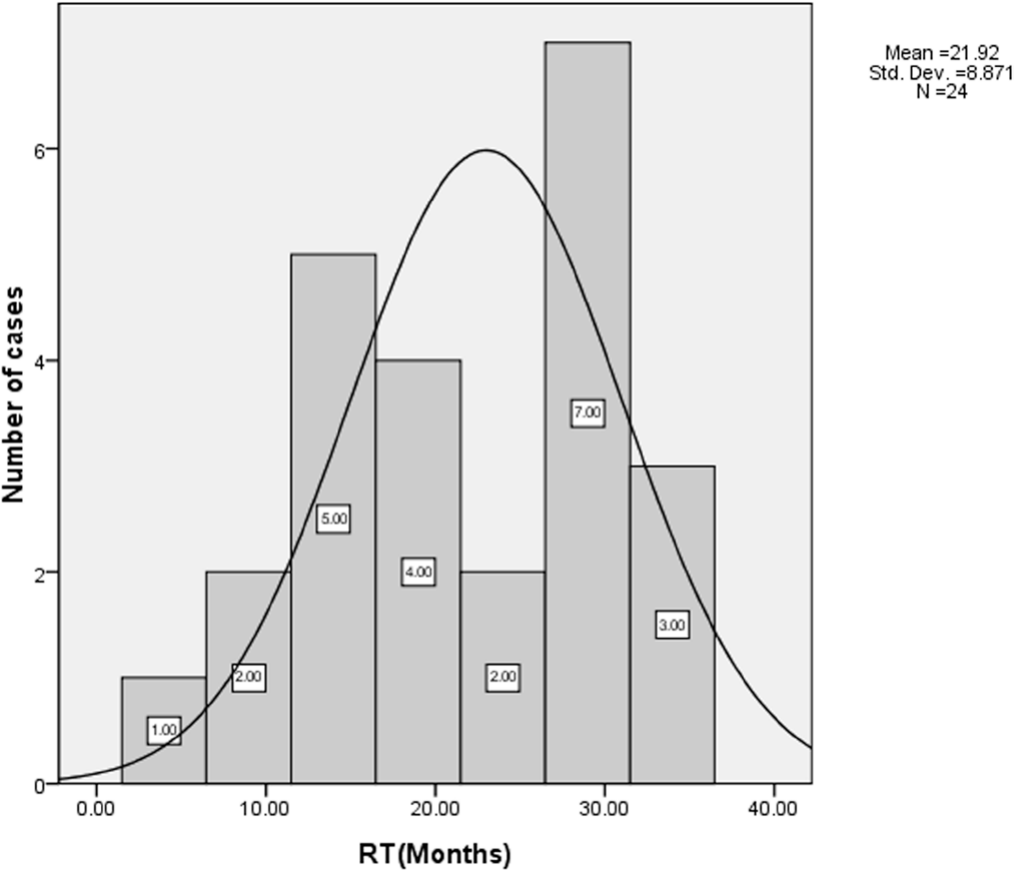
Histogram of number of recurrent cases and their recurrence time. The bimodal distribution of recurrence time (RT) indicated that 12 months and 30 months after primary pterygium resection were two risk times for pterygium recurrence, which might involve different underlying mechanisms.

There were 24 cases of pterygium recurrence. The recurrence rate (RR) of Grade 1 was 28.1% (9/32), Grade 2 was 24.1% (7/29), and Grade 3 was 22.9% (8/35), as classified in the primary pterygium (without significant difference between the three groups); the overall RR was 25% (24/96) for all patients during the three-year follow-up. The average RT was 21.9 months (standard deviation=8.87). A bimodal distribution of RT was found (Figure 3), indicating that 12 months (5/24) and 30 months (7/24) after primary pterygium resection were two risk times for pterygium recurrence, which might involve two different underlying mechanisms.

### Clinicopathologic correlation

**Figure 4:**
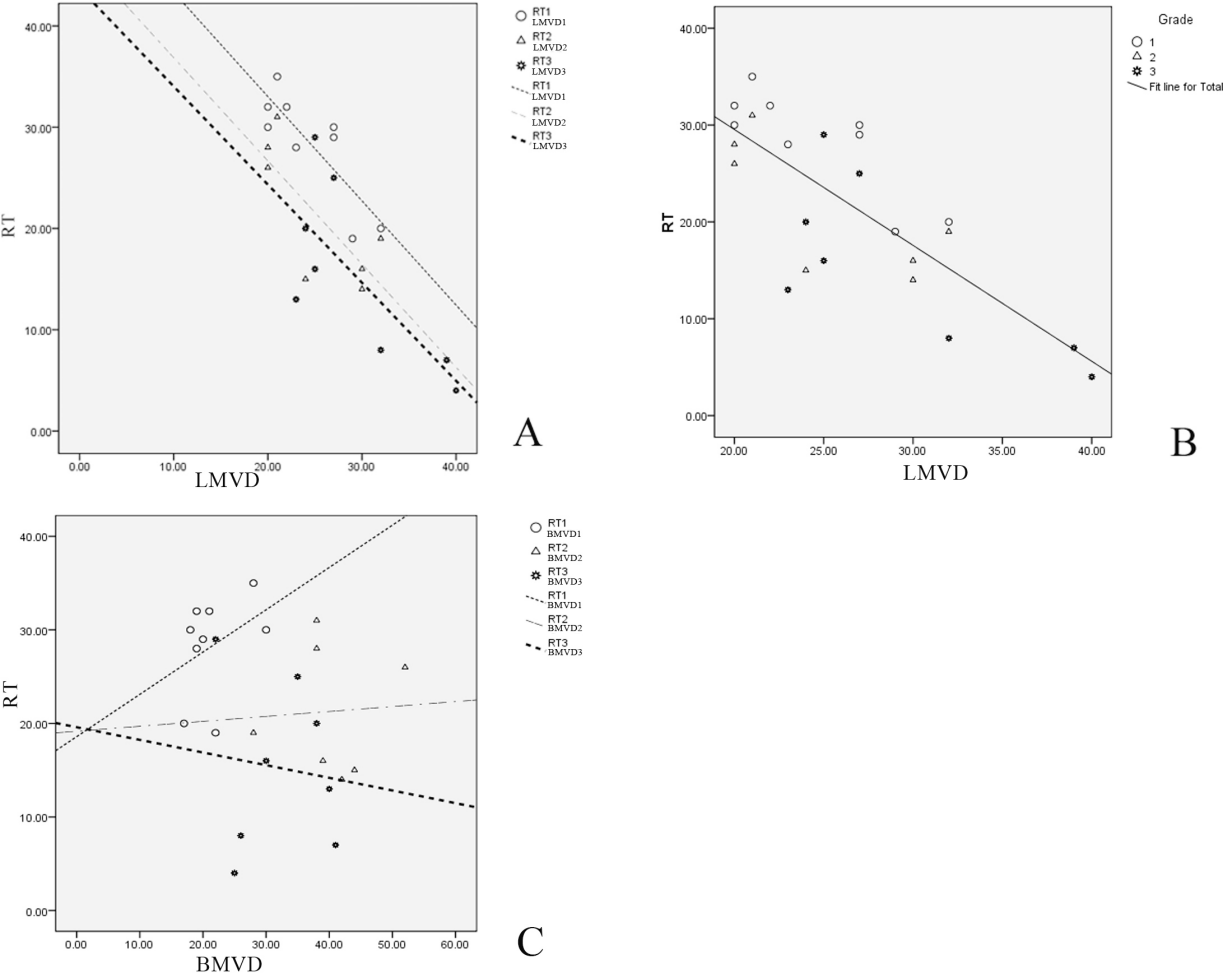
The relationship between lymphatic microvessel density, blood microvessel density, and recurrence time in different recurrent cases. **A**: Scatter distribution of recurrence time (RT) in different grades of pterygium was with best fit lines for lymphatic microvessel density (LMVD) in each grade. **B**: Scatter distribution of RT in all cases was with a fit line for total LMVD. **C**: Scatter distribution of RT in different grades of pterygium was with best fit lines for blood microvessel density (BMVD) in each grade, but the fit line for total BMVD did not exist.

The relationship between RT, hemangiogenesis, and lymphangiogenesis in different grades of pterygium in our study is shown in Figure 4. Using the RT and the immunochemical analysis of LMVD and BMVD, we statistically analyzed the relationship between angiogenesis and recurrence time to determine which one (lymphatic or blood vessels) played a more important role in accelerating pterygium recurrence. Paired Student *t* tests were used to compare the correlation between RT and LMVD, BMVD, or both. The results show that RT significantly correlated with LMVD (correlation=–0.577, p=0.004) but not with BMVD or (LMVD+BMVD). Depending on our statistical analysis, we demonstrated that the recurrence time of pterygium was negatively correlated with lymphangiogenesis (LMVD) in different grades of pterygium (Figure 4A) and in total cases (Figure 4B), and an LMVD greater than 20 in the surgical specimens predicted pterygium recurrence. However, there was a best fit line for the distribution of RT and BMVD in each grade of pterygium (Figure 4C), but the fit line for total BMVD did not exist.

## Discussion

Due to high prevalence and surgery recurrence characteristics, pterygium has always been a major concern to ophthalmologists [2]. As reported in the literature, the RR for simple pterygium excisions can be as high as 30% to 69% [31]. Therefore, effective treatment of pterygium is an important part of clinical ophthalmology and basic research. With the development of eye microsurgery, the RR of pterygium surgery has been greatly reduced. In our study, an overall pterygium RR of 25% was observed for all patients during the three-year follow-up, which highlights the development of improved treatments for pterygium.

However, development in clinical surgical techniques falls short of meeting the needs of the increasing number of patients with pterygium, and ultimately, solving the problem requires inhibiting pterygium pathogenesis. The pathogenesis of pterygium is still not clear [32], despite various hypotheses, including those involving apoptosis, limbal stem cell deficiency [31], viral infection [33], matrix metalloproteinases [34], and the immune system [35,36]. However, the lack of good research data has prevented further progress in treating pterygium [37].

Progress in identifying specific lymphatic endothelium markers [38] has improved our understanding of lymphangiogenesis in the pathogenesis of many diseases, including pterygium. In our previous study [16], we applied enzyme histochemistry to demonstrate that gray-stained lymphangiogenesis occurs rarely in normal conjunctival and quiescent pterygium tissues but is widely distributed in pterygium tissue of advanced and recurrent stages. Furthermore, there were statistically significant differences between the research groups (advanced and recurrent phase) and the control group, which confirmed the results of Aspiotis’s report [39]. In our other later study [17] of pterygium using LYVE-1 and CD31 double immunohistochemistry, we distinguished lymphatic vessels from blood vessels, studied new lymphangiogenesis, and demonstrated that transient upregulation of vascular endothelial growth factor-C may be responsible for the development of lymphatic vessels in pterygia. For further study, we conducted this prospective case series study and sought to provide additional clinical evidence for our hypothesis that abnormal conjunctiva (lymph) angiogenesis is involved in the pathogenesis and accelerated recurrence of pterygium.

In the current study, we divided patients with pterygium into three groups and examined (lymph) angiogenesis in each excised specimen. Compared to normal conjunctiva, all grades of pterygium presented with statistically significant higher average count of microvessels (BMVD and LMVD) compared to normal conjunctiva (Table 2). Although we are the first to report the LMVD in different groups of pterygium and normal conjunctiva, Aspiotis et al. [39] reported that pterygium presented with a statistically significant higher average count of microvessels (BMVD) compared to normal conjunctiva (17.97±8.5 versus 5.72±5 per high-power field, p=0.001), which was much lower than our study. The different methods for detecting and quantifying BMVD at different power fields of microscopy and the different grades of pterygium included (the grades of pterygium were not mentioned in Aspiotis’s study) in different races in the two studies might contribute to this variation. However, the BMVD and LMVD results were consistent with our previous studies in different sets of patients [16,17]. In the current study, despite the significant relationship between BMVD and LMVD, the new findings were that LMVD were mildly increased in Grade 1 pterygia but were dramatically increased in Grades 2 and 3 pterygia (Table 2, all p values <0.01). However, relative to LMVD, there were greater blood vessel densities in Grade 2 pterygia, but the BMVD decreased in Grade 3. Our new finding might suggest that LMVD is a more suitable marker for the severity of pterygiums than BMVD, although more studies are needed to make this conclusion.

The strengths of the present study are to determine whether LMVD and/or BMVD is related to the accelerated recurrence of pterygia, and whether they could be used as predictive markers for the recurrence time of pterygia. In a previous report by Džunić et al. [40], they used the classical histochemical methods to pathologically analyze the specimens taken from 55 patients, and they found a positive correlation with pathological blood vessels (BMVD) and pterygium recurrence. However, in the current study, we used more advanced immunohistochemistry to analyze the clinicopathologic correlation in a larger population (96 cases) of pterygium, resulting that pterygium RT was negatively correlated with LMVD (p<0.05) but not BMVD or the preoperative grades of pterygium. Moreover, there was no statistical difference between the RR in different preoperative grades of pterygium in our study. We supposed that RR not only depended on LMVD but was also multifactor, and RT was more suitable to be used as a factor representing pterygium recurrence than RR. Interestingly, a bimodal distribution of RT was found in our study, indicating that 12 months and 30 months after primary pterygium resection were two risk times for pterygium recurrence, which might involve two different underlying mechanisms. Therefore, LMVD might be one of the contributing factors of pterygium recurrence, and LMVD could accelerate the recurrence. In other words, the more LMVD is detected, the shorter the RT if pterygium recurrence is doomed to happen. The difference in surgical techniques and postoperative protocol may contribute to the conflicting result between our study and Džunić et al.’s study [40]. To our knowledge, this is the first time that the relationship between lymphatic microvessel density and pterygium recurrence time has been documented, although there is still no clear evidence how lymphangiogenesis leads to the recurrence of pterygium. Whether lymphangiogensis itself or an immunological process associated with lymphangiogenesis indirectly contributes to the pterygium proliferation and/or invasion (or conjunctival tissue proliferation) in vitro requires further research.

Despite the limitations, the current study remains one of the first to determine the relationship of RT and BMVD/LMVD. Although RT was not correlated with BMVD or pterygium grade, LMVD was significantly correlated with overall RT and RT in each group of patients. Additionally, an LMVD greater than 20 in the surgical specimens predicted pterygium recurrence. We conclude that the lymphatic microvessel density of surgical specimens is an independent risk factor and a valuable predictive factor for the recurrence time of pterygium.
